# Incidence of Glucose-6-Phosphate Dehydrogenase Deficiency among Swedish Newborn Infants

**DOI:** 10.3390/ijns5040038

**Published:** 2019-10-29

**Authors:** Annika Ohlsson, Katarina Rehnholm, Kumar Shubham, Ulrika von Döbeln

**Affiliations:** 1 Centre for Inherited Metabolic Diseases, Karolinska University Hospital Solna, SE-171 76 Stockholm, Sweden; katarina.rehnholm@sll.se (K.R.); ulrika.vondobeln@sll.se (U.v.D.); 2 Department of Medical Biochemistry and Biophysics, Division of Molecular Metabolism, Karolinska Institute, SE-171 77 Stockholm, Sweden; 3 Department of Anesthesiology and Intensive Care, Mälarsjukhuset, SE-631 88 Eskilstuna, Sweden; 4 LabSystems Diagnotics Oy, Tiilitie 3, FIN-01720 Vantaa, Finland; kumar.shubham@labsystemsdx.com

**Keywords:** glucose-6-phosphate dehydrogenase deficiency, newborn screening, mutations

## Abstract

Sweden has 10.2 million inhabitants and more than 2.4 million have a foreign background. A substantial number of immigrants come from countries where glucose-6-phosphate dehydrogenase deficiency (G6PDD) is frequent. The total birth rate annually in Sweden is approximately 117,000 and newborn screening is centralized to one laboratory. We determined glucose-6-phosphate dehydrogenase (G6PD) activity in 10,098 dried blood spot samples (DBS) from the whole country with a fluorometric assay (LabSystems Diagnostics Oy, Finland). The first 5451 samples were anonymised and run as singletons, whilst the following 4647 samples were coded. Enzyme activity ≤40% of the mean of the day was found in 58 samples (1/170) and among these, 29 had activities ≤10% (1/350). Twenty-nine samples with residual activities between 2–39% in the coded cohort were subjected to Sanger sequencing. Disease-causing variants were identified in 26 out of 29 infants, of which six were girls. In three patients, we did not find any disease-causing variants, although two patients were hemizygous for the known polymorphisms c.1311T>C and c.1365-13C>T. The most common disease-causing variant found in 15 of the 29 samples (12 hemizygotes, two heterozygotes, one homozygote) was the Mediterranean mutation, c.563C>T (p.(Ser188Phe)) in exon 6. G6PDD is thus a surprisingly prevalent disorder in Sweden.

## 1. Introduction

Glucose-6-phosphate dehydrogenase (G6PD, E.C.1.1.1.49) is a key enzyme in the pentose phosphate pathway, which produces ribose-5-phosphate. G6PD is extremely important for the reduction of nicotinamide adenine dinucleotide phosphate (NADP^+^) to NADPH + H^+^. It is a housekeeping enzyme important for the control of oxidative stress, especially in red blood cells, which lack nucleus and mitochondria. Patients with glucose-6-phosphate dehydrogenase deficiency (G6PDD, OMIM_305900) are asymptomatic unless they have a very low residual activity or are exposed to some specific triggers, including certain medicines, infections or fava beans (*Vicia faba*), the latter being the most common trigger [1]. The clinical presentation of the disorder is dependent on the level of residual activity of the enzyme and the load of the trigger. Older children and adults can develop acute haemolytic anaemia, whilst newborn infants are at risk of severe jaundice and kernicterus. There is no cure for the disorder, but once diagnosed, the patients can avoid known triggers and be treated promptly when they get symptoms.

The disorder has a continuum of severity of the disease and a complete absence of enzyme activity is considered not compatible with life [2]. G6PDD is commonly divided into four classes based on the residual activity of the enzyme [3], as shown in Table 1.

The disease was discovered in 1956 [4] and is traditionally known to be common in the Mediterranean region, Africa, the Middle East and Asia, but with migration, it is now present worldwide. It is the most common human genetic enzymopathy, estimated to affect more than 300 million people or 5% of the global population, as published in 2008 [5]. G6PDD is correlated to protection against the malaria parasites *Plasmodium falciparum* and *Plasmodium vivax* [6], and this probably explains why it is widespread in regions where the mosquitos harbouring these parasites thrive. In the past decade, G6PDD has generated interest, since it appears to have an impact on disorders like cardiovascular disease, diabetes, kidney disease, colorectal cancer and sepsis [7,8,9,10,11,12]. One reason for that is that G6PD is the rate-limiting enzyme of the pentose phosphate pathway as well as a source of NADPH, which is crucial in the intermediary metabolism and cell survival [13].

### 1.1. Newborn Screening

As reported in an overview of newborn screening (NBS) worldwide, from 2015, general screening of newborn infants for G6PDD had only been implemented in a few places. This includes a couple of states in the United States of America, Greece, Panama and six countries in the Asia Pacific, with pilot screening programmes in an additional nine countries [14]. The incidence of G6PDD in the northern part of Europe is unknown. We know of one published Nordic study reporting the prevalence of G6PDD, and this was among 1500 adult immigrants (of non-northern European origin) in Denmark. In this cohort, the calculated allele frequency for G6PDD was estimated to between 2.4 and 2.9% [15]. Ethical permission for the study was given by the Regional Ethical Committee in Stockholm, 2019/991-31/2.

### 1.2. The G6PD Gene

The *G6PD* gene (NG_009015.2) is located on chromosome Xq28 and is 18 kb long and consists of 13 exons and 12 introns (exon 1 being non-coding) and codes for a protein of 514 amino acids [16]. Most disease-causing variants are either missense or nonsense, the latter only found in heterozygous females since the complete loss of enzyme activity is considered not to be compatible with life [2]. Disease-causing variants have often been named after the geographic area where they are common, for example the “Mediterranean” variant, c.563C>T (p.(Ser188Phe)) [17].

### 1.3. Aim

Sweden has 10.2 million inhabitants and more than 2.4 million have a foreign background (born abroad or both parents born abroad). A substantial number of immigrants come from countries where G6PDD is frequent. The total birth rate annually in Sweden is approximately 117,000, and the NBS is centralized to one laboratory. The aim of this study was to investigate the incidence of G6PDD in Sweden by analysis of 10,000 blood samples taken in a program screening infants in the neonatal period.

## 2. Materials and Methods

**Figure 1:**
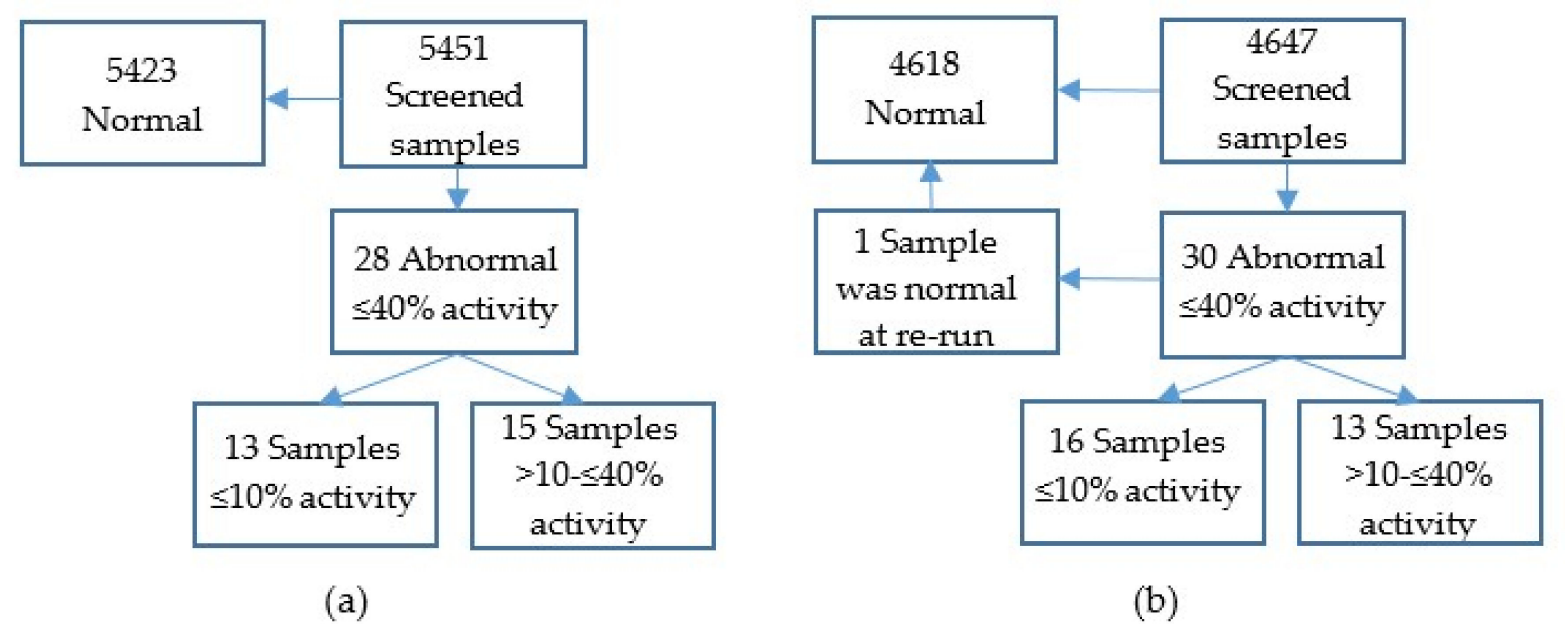
(**a**) Summary of results for samples screened anonymously and analysed as singletons. (**b**) Summary of results for samples screened coded. Abnormal samples were re-analysed in duplicate. Samples with residual enzyme activities ≤40% were subjected to Sanger sequencing.

Included in the study were 10,098 fresh samples from newborn infants from the whole country. They were screened for two periods of three weeks. Initially, 5451 samples were analysed as singletons to test if a recall level, empirically set at a residual activity of ≤40%( Figure 1a), would result in a reasonable amount of samples for further analysis. Then, 4647 coded samples were analysed (Figure 1b), which enabled a re-run of samples below the cut-off in duplicate, in accordance with the routine for disorders included in the Swedish screening program, followed by genetic analyses of samples with a confirmed low activity.

**Scheme 1:**
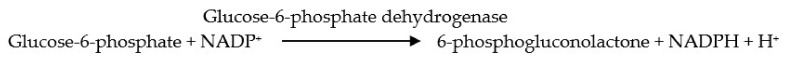
The principle of fluorometric measurement of G6PD activity.

Fluorometric determination of G6PD activity from blood specimens dried on filter paper (dried blood spots (DBS)) was performed with the Neonatal G6PD, fluorometric test kit 6199860 (LabSystems Diagnostics Oy, Vantaa, Finland). The quantitation of the product of the enzymatic reaction was performed with excitation at wavelength 355 nm and emission at 460 nm. The assay measures the formation of NADPH, when NADP^+^ is reduced by the G6PD enzyme in the presence of glucose-6-phosphate (Scheme 1).

Enzyme activity was expressed as percent residual activity of the mean of the samples analysed on the same day and as U/gHb. Cut-off for re-run was set at ≤40% activity of the mean, corresponding to ≤3.5 U/g Hb. The cut-off was chosen to ensure that all infants with a high risk of developing symptoms were identified. 

DNA was extracted from two DBS with a diameter of 3.2 mm with QIAmp DNA Micro Kit (QIAGEN, Venlo, The Netherlands). Exons 2–13 were amplified with PCR in nine fragments, followed by Sanger sequencing of all exons and exon/intron boundaries. Direct-cycle sequencing of all PCR fragments was performed with a BigDye Terminator v3.1 Cycle Sequencing kit (Applied Biosystems, Foster City, CA, USA) following the manufacturer’s recommendations. We have not searched for variants in the 5′ or the 3′ UTR region or for rearrangements or deletions.

## 3. Results

**Figure 2:**
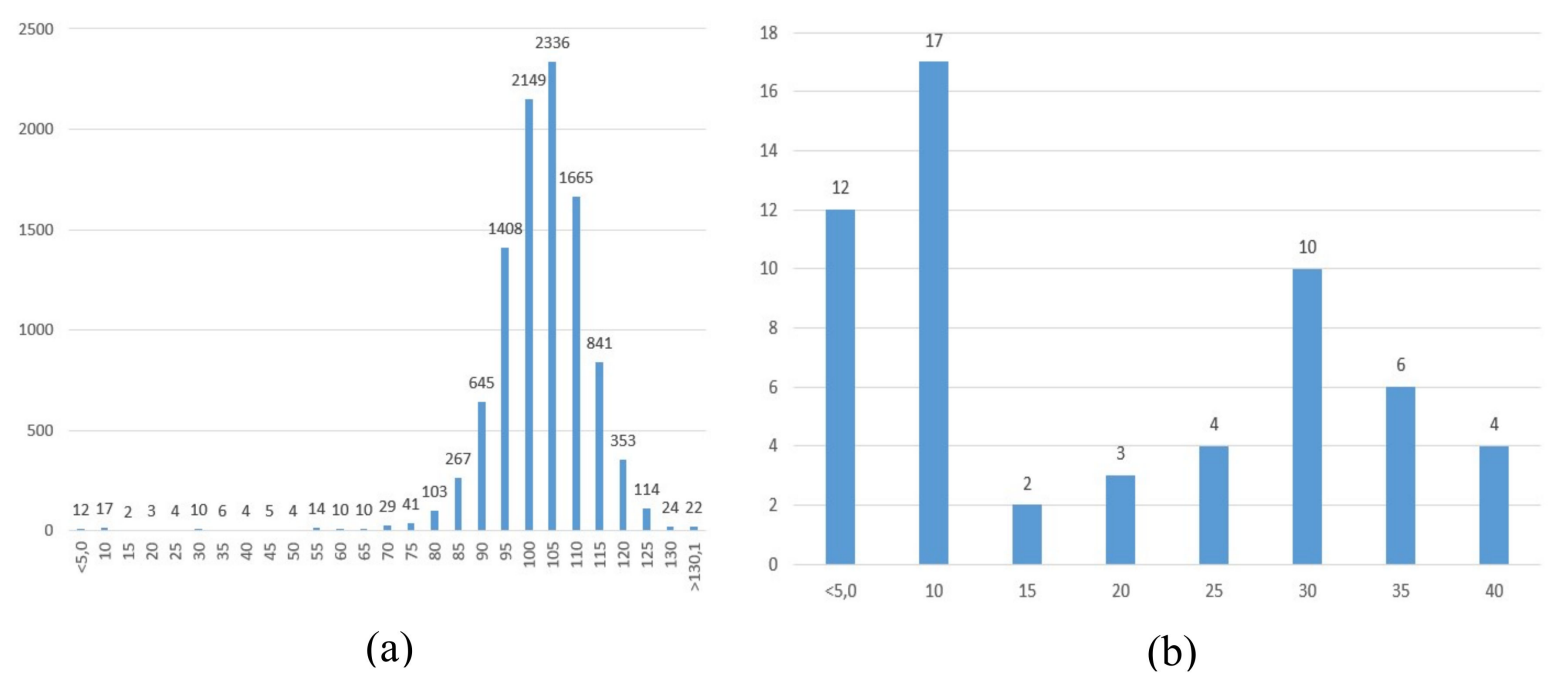
(**a**) Distribution of glucose-6-phosphate dehydrogenase activity in 10,098 screened samples, expressed as percent of the days mean. (**b**). Distribution of glucose-6-phosphate dehydrogenase activity in samples (*n* = 58) with ≤40% residual activity, expressed as percent of the days mean, analysed as singletons.

The mean G6PD activity of the 10,098 samples was 8.7 U/gHb. Fifty-eight samples had G6PD residual activities below the cut-off (≤40%), incidence 1/170, and of these, 29 had a residual activity of ≤10%, incidence 1/350 (Figure 1a,b and Figure 2a,b).

In the coded cohort (Figure 1b), ten variants in the *G6PD* gene were detected in 29 infants, including the two putatively benign variants c.1311T>C (p.(Tyr437Tyr)) and c.1365-13C>T. The latter variants have been considered polymorphisms but there are several articles describing associations with low G6PD enzyme activity in patients carrying the c.1311T>C, c.1365-13C>T and the 3′ UTR c.*+357A>G variant without other variants in the *G6PD* gene [18,19]. An intronic variant, possibly causing aberrant splicing, c.267+5G>A, not described before, was found on one allele. It was detected in a patient who was compound heterozygous for c.[267+5G>A];[563C>T] with residual activity of 8% of normal. In one patient with ≤10% activity, we could not detect any disease-causing variants or the two polymorphisms by Sanger sequencing. The most common disease-causing variant, found on 16 alleles, was c.563C>T (p.(Ser188Phe)), also known as the “Mediterranean” variant (Table 2).

Of the 29 samples which were sequenced, six were from girls (21%). Two of the girls had a G6PD activity ≤10% of normal, which represents 12.5% (2/16) of the infants in severity class II, as compared to 31% (4/13) being girls in class III (Table 3).

All but two infants (88%, (14/16)) in severity class II were either hemizygous, heterozygous or homozygous for c.563C>T, in contrast to heterogeneity of genotypes present in class III with only one patient being compound heterozygous for c.[563C>T];[=]. In three patients, we did not find any disease-causing variants, although two were hemizygous for the known polymorphisms c.1311T>C and c.1365-13C>T (Table 3).

## 4. Discussion

The incidence of G6PDD in Sweden was unknown before this study. Our results indicate that 1/350 newborn infants have a G6PDD with an enzyme activity of ≤10% of normal. With the present annual number of births in Sweden of 117,000, approximately 330 patients would be recalled with a suspected severe G6PDD if newborn screening for G6PDD was to be implemented in the Swedish NBS programme. If all newborn infants with residual activity of ≤40% of normal were to be recalled, this number would increase to almost 700 or 0.6% of all newborn infants.

The proportion of boys and girls among the 58 infants with an enzyme activity below the cut-off (≤40%) in this study is unknown. NBS screening programmes detect hemizygous boys and homozygous girls, missing out most of the heterozygous girls [20]. In the genetic study, five girls were either homozygous or compound homozygous for disease-causing variants. Only one girl was heterozygous for one severe disease-causing variant. Heterozygous girls would probably be detected by increasing the cut-off to ≤65% of the days mean G6PD activity. This would almost double the number of recalls (Figure 2a). If the goal is to detect infants with severe G6PDD and at risk of developing chronic non-spherocytic haemolytic anaemia or intermittent haemolysis, severity class I and II, the cut-off can be left at ≤40%. If the proportion of girls with G6PDD in Swedish neonates with a G6PD activity of ≤40% is true, the disorder seems to affect almost 0.5% of all newborn boys in Sweden if the birth rate is equal between genders.

With the immigration to Sweden from areas in the world with a known high prevalence of G6PDD, it is not surprising that the incidence of newborn infants with an enzyme activity less or equal to 40% is as high as 1/170. This is much higher than indicated by clinically diagnosed patients. In a recent article where the incidence of hazardous hyperbilirubinaemia (1/15,000) and kernicterus (1.3/100,000) in newborn infants was studied, the authors conclude that the incidence of the two diagnoses is increasing [21]. This is an important discussion, as one could speculate that when G6PDD is becoming more common, it may lead to an increase in the incidence of kernicterus and haemolytic anaemia in the neonatal period. Kernicterus was almost unknown (in modern times) before early discharge (before 24 h of age) after delivery was implemented in Sweden: too early for jaundice to have developed. If patients with G6PDD develop hyperbilirubinaemia, it is of great importance that these infants are handled correctly to avoid kernicterus followed by complications.

Screening for G6PDD has been recommended if the incidence in newborn boys is more than 4–5% [3]. This is not the case in Sweden with an incidence of approximately 0.5%, but it may well be increasing. When the increasing number of immigrants have babies, G6PDD could be one factor behind the increasing incidence of kernicterus in Sweden [20]. In comparison to the disorders currently included in the Swedish NBS programme, the incidence of G6PDD in Sweden is very high. The fact that only a minority of the patients will ever exhibit symptoms has to be taken into consideration when screening for G6PDD is discussed. It is also doubtful if the results from the NBS test will be available in time for the start of treatment to prevent the development of hazardous hyperbilirubinaemia during the first week of life, since the results are usually not ready before six days of age. The early diagnosis would, however, be of benefit for parents to be able to avoid triggers for children at risk. 

G6PDD is becoming a more common disorder in Sweden. It is important for clinicians to be aware of the disorder when patients originating from areas where malaria was or still is common and G6PDD frequent, are diagnosed with acute haemolytic anaemia. This is also the case when newborn infants develop hyperbilirubinaemia rapidly or develop hyperbilirubinaemia not responding to traditional treatments. NBS for G6PDD ought to be kept in mind in the future since an increase in the incidence among newborn infants is to be expected.

## Figures and Tables

**Table 1:** Classification of glucose-6-phosphate dehydrogenase deficiency (G6PDD) with respect to glucose-6-phosphate dehydrogenase (G6PD) activity.

Class	Enzyme Activity	Severity
I	<1%	Severe with chronic non-spherocytic anaemia
II	1–10%	Severe with intermittent haemolysis
III	10–60%	Mild to moderate haemolysis triggered by stressors only
IV	60–150%	Normal activity

**Table 2:** *Glucose-6-phosphate dehydrogenase* gene variants detected in the screened cohort.

Exon/ Intron	Nucleotide Change	Predicted Effect on Protein	dbSNP	Classi- Fication	Number of Alleles
Exon 2	c.95A>G	p.(His32Arg)	rs137852340	II	1
Exon 4	c.202G>A	p.(Val68Met)	rs1050828	III	4
Intron 4	c.267+5G>A	p.(?)	Novel	II	1
Exon 5	c.376A>G	p.(Asn126Asp)	rs1050829	I–IV	2
Exon 6	c.563C>T	p.(Ser188Phe)	rs5030868	II	16
Exon 9	c.871G>A	p.(Val291Met)	rs137852327	III	2
Exon 9	c.969T>C	p.(Leu323Pro)	rs76723693	III	2
Exon 11	c.1311T>C	p.(Tyr437Tyr)	rs2230037	III (SNP)	15
Exon 11	c.1360C>T	p.(Arg454Cys)	rs398123546	II	1
Intron 11	c.1365-13C>T	p.(?)	rs2071429	III (SNP)	6

dbSNP, Single Nucleotide Polymorphism Database; SNP, Single Nucleotide Polymorphism.

**Table 3:** Classification and genotypes of newborn infants with activity ≤40% of the days mean.

Pat	Class	G6PD-Activity ^1^	Nucleotide Change	Predicted Effect on Protein
**01**	II	2%	c.[1360C>T];[0]	p.[(Arg454Cys)];[0] ^6^
**02**	II	4%	c.[563C>T];[0]	p.[(Ser188Phe)];[0]
**03**	II	4%	c.[563C>T];[0]	p.[(Ser188Phe)];[0]
**04**	II	4%	c.[563C>T];[0]	p.[(Ser188Phe)];[0]
**05**	II	5%	c.[563C>T];[0]	p.[(Ser188Phe)];[0] ^3^
**06**	II	5%	c.[563C>T];[0]	p.[(Ser188Phe)];[0]
**07**	II	6%	c.[563C>T];[0]	p.[(Ser188Phe)];[0]
**08**	II	6%	c.[563C>T];[0]	p.[(Ser188Phe)];[0] ^3^
**09**	II	7%	c.[563C>T];[0]	p.[(Ser188Phe)];[0] ^3,6^
**10**	II	7%	c.[563C>T];[0]	p.[(Ser188Phe)];[0]
**11**	II	7%	c.[563C>T];[563C>T]	p.[(Ser188Phe)];[(Ser188Phe)] ^2^
**12**	II	8%	c.[563C>T];[0]	p.[(Ser188Phe)];[0]
**13**	II	8%	c.[267+5G>T];[563C>T]	p.[?];[(Ser188Phe)] ^2,4,7^
**14**	II	9%	N.D	p.[=];[0]
**15**	II	10%	c.[563C>T];[0]	p.[(Ser188Phe)];[0]
**16**	II	10%	c.[563C>T];[0]	p.[(Ser188Phe)];[0]
**17**	III	14%	c.[95A>G];[0]	p.[(His32Arg)];[0]^6^
**18**	III	14%	c.[871G>A];[0]	p.[(Val291Met)];[0]
**19**	III	18%	c.[406C>T;1048G>C];[0]	p.[(Arg136Cys;Asp350His)];[0]
**20**	III	20%	c.[376A>G;524A>T;968T>C];[376A>G]	p.[(Asn126Asp;Asp181Val;Leu323Pro)];[(Asn126Asp)] ^2,5^
**21**	III	22%	c.[871G>A];[0]	p.[(Val291Met)];[0]
**22**	III	22%	c.[202G>A;376A>G];[0]	p.[(Val68Met;Asn126Asp)];[0] ^3^
**23**	III	23%	c.[202G>A;376A>G];[0]	p.[(Val68Met;Asn126Asp)];[0] ^3^
**24**	III	24%	N.D	p.[=];[0] ^3,6^
**25**	III	25%	c.[376A>G;968T>C];[0]	p.[(Asn126Asp;Leu323Pro)];[0] ^3^
**26**	III	25%	c.[202G>A;376A>G];[202G>A;376A>G]	p.[(Val68Met;Asn126Asp)];[(Val68Met;Asn126Asp)] ^2,5^
**27**	III	36%	c.[202G>A;376A>G];[202G>A;376A>G]	p.[(Val68Met;Asn126Asp)];[(Val68Met;Asn126Asp)] ^2,5^
**28**	III	37%	c.[563C>T];[=]	p.[(Ser188Phe)];[=] ^2^
**29**	III	39%	N.D	p.[=];[0] ^3,6^

^1^ Mean activity of 3 determinations, ^2^ Girl, ^3^ Hemizygous for c.1311T>C, ^4^ Heterozygous for c.1311T>C, ^5^ Homozygous for c.1311T>C, ^6^ Hemizygous for c.1365-13C>T, ^7^ Heterozygous for c.1365-13C>T, Not detected, N.D.
